# Development of Dispersive Liquid–Liquid Microextraction Method Based on Solidification of Floating Organic Droplets for Rapid Determination of Three Strigolactones in Rice (*Oryza sativa* L.) Using Ultra-High-Performance Liquid Chromatography–Tandem Mass Spectrometry

**DOI:** 10.3390/ijms26094337

**Published:** 2025-05-02

**Authors:** Xianxin Zhu, Zihan Wu, Xunzhi Deng, Ze Liao, Ruozhong Wang, Zhoufei Luo

**Affiliations:** 1 College of Bioscience and Biotechnology, Hunan Agricultural University, Changsha 410128, China; zhuxianxinstudent@126.com (X.Z.); wuzihan@stu.hunau.edu.cn (Z.W.); dxzhn2002@163.com (X.D.); lz602174935@gmail.com (Z.L.); 2 Hunan Provincial Key Laboratory of Phytohormones and Growth Development, Hunan Agricultural University, Changsha 410128, China

**Keywords:** strigolactones, rice, dispersive liquid–liquid microextraction based on solidification of floating organic droplets (DLLME-SFO), UHPLC-MS/MS

## Abstract

Strigolactones (SLs) are key hormones regulating branching and tillering in rice, impacting plant architecture and yield. A rapid, sensitive, and environmentally friendly method using dispersive liquid–liquid microextraction based on the solidification of floating organic droplets (DLLME-SFO), coupled with ultra-high-performance liquid chromatography and tandem mass spectrometry (UHPLC-MS/MS), has been developed for the determination of three SLs (strigol, orobanchol, and 5-deoxystrigol). The DLLME-SFO method integrates one-step low-temperature extraction and enrichment. The DLLME-SFO conditions were optimized through a single-factor experimental design. Under the best-tested conditions, the developed method exhibited excellent linearity, with the coefficient of determination (R^2^) values greater than 0.9993. The recoveries ranged from 83% to 96%, with precision values ranging from 4.5% to 12.4%. The limits of detection (LODs) varied from 0.6 to 1.2 pg/g fresh weight, indicating the high sensitivity of the method. Additionally, a novel assay protocol for the quantification of SLs in rice in response to nitrogen and phosphorus stress conditions was applied.

## 1. Introduction

Strigolactones (SLs) are plant hormones of various plant physiological processes including shoot branching, root architecture, root morphology, and stress responses [1]. SLs are primarily synthesized in the root tissues of plants and transported via the xylem to axillary buds, where they modulate bud growth and development [2]. Natural SLs can be classified into two groups according to the orientation of the C-ring. The first group comprises strigol-type SLs, which contain a *β*-oriented C-ring and include compounds like strigol acetate, sorgomol, sorgolactone, strigone, and 5-deoxystrigol (5-DS). The second group comprises orobanchol-type SLs, which are characterized by an *α*-oriented C-ring and include compounds such as orobanchol acetate, fabacyl acetate, 4-deoxyorobanchol(4-DO), and solanacol [3]. Strigol, the first identified SL, was extracted from the root exudates of cotton plants [4]. 5-DS was identified as a common precursor of SLs due to its structural simplicity in 2005 [5]. Orobanchol is currently the most widely distributed form of SL in higher plants [6]. These three SLs (strigol, orobanchol, and 5-DS) are of considerable significance in agricultural production and have garnered substantial research interest.

The analysis of SLs is particularly challenging due to their exceptionally low endogenous concentrations in plant tissues, typically measured in picograms per gram of fresh weight [7]. For instance, only 70 picograms of orobanchol per plant have been detected in red clover seedling roots [8]. This characteristic of extremely low natural abundance in plant organisms renders their extraction from botanical sources both technically challenging and economically prohibitive. Current analytical methods, such as liquid chromatography–mass spectrometry (LC-MS), often struggle with sensitivity and specificity, especially in complex plant matrices where interfering compounds may co-elute [9]. SLs exhibit significant instability, rendering them highly susceptible to degradation, especially via nucleophilic attack targeting the enol ether bond between the SL rings [10]. This enol ether bond, being unsaturated and chemically labile, contributes to the overall instability of SLs, making them particularly sensitive to environmental factors such as light, heat, and hydrolysis [11]. Additional challenges in SL analysis arise from the high complexity of plant matrices, as well as from the limited chemical, photostability, and thermal stability of many target compounds [12]. This is compounded by the lack of standardized protocols for SL extraction and purification across different plant tissues [13]. Thus, developing analytical methods that consider the physiological properties of SLs remains a significant challenge.

Rice (*Oryza sativa* L.), one of the three main food crops, is a staple food for more than half of the world’s population, and increasing its yield is the primary goal of breeding [14]. SLs that are involved in rice tiller regulation have become essential tools for controlling plant architecture and improving yield [15]. Nutrient deficiency represents a significant abiotic stress factor, prompting plants to adopt various survival strategies, including enhancing nutrient uptake efficiency and establishing symbiotic relationships with beneficial microorganisms [16]. The synthesis of SLs in rice is influenced by nitrogen (N) and phosphorus (P) starvation. Compared with those under P starvation conditions, the 4-DO levels of wild-type (Nipponbare) seedlings increased over 300-fold in root exudates and 600-fold in root tissues [17]. Therefore, to better understand the molecular mechanisms and signaling pathways of SLs and their precise roles in complex regulatory networks, it is of significance to investigate the distribution of SLs in rice tillering research.

Sample preparation is an important step in most analytical procedures as it helps minimize matrix effects and concentrate the target analytes. Dispersive liquid–liquid microextraction (DLLME) is a technique derived from traditional liquid–liquid extraction. In DLLME, a mixture of extractant and dispersant is introduced into the aqueous sample. The extractant rapidly forms small droplets that disperse throughout the aqueous phase, significantly improving extraction efficiency [18]. Dispersive liquid–liquid microextraction based on the solidification of floating organic droplets (DLLME-SFO) is a more recent variant of DLLME. In this method, the floating solvent used for extraction can be easily and quickly recovered from the aqueous phase after solidifying at low temperatures [19]. Due to its easy operation, high sensitivity, and reduced consumption of organic solvents, DLLME has gained popularity as a sample preparation method [20]. DLLME-SFO is a promising method for the analysis of plant hormones. Currently, it has not been applied to the determination of trace plant hormones in plant samples.

Currently, ultra-high-performance liquid chromatography–tandem mass spectrometry (UHPLC-MS/MS) is a leading analytical tool that enables the efficient separation and high-sensitivity detection of SLs [21]. However, because SLs are neutral lactones and lack easily ionizable functional groups, co-existing matrix components that are easily ionized can suppress their ionization, leading to decreased mass spectrometric sensitivity [22]. Therefore, optimizing the mass spectrometric conditions for SLs is crucial for their accurate quantification.

Thus, the aims of this study were (1) to develop a method for the analysis of three target SLs (strigol, orobanchol, 5-DS) from rice samples using DLLME-SFO followed by UHPLC-MS/MS analysis, (2) to optimize the DLLME-SFO parameters by performing a series of trials to validate the quantification of SLs in rice roots, and (3) to determine SL levels in rice under nitrogen and phosphorus stress conditions. This novel method could be applied to the analysis of SLs in various plant species.

## 2. Results and Discussion

### 2.1. The Best Tested Conditions of the DLLME-SFO Parameters

**Figure 1:**
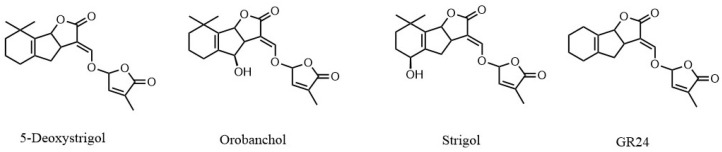
The structures of the three SLs and GR24.

The DLLME-SFO method was tested, including the selection of suitable extractants and dispersants, the determination of the best tested volumes, pH adjustment, and the exploration of potential benefits from ultrasonic assistance. To test the method, a 200 mg sample of blank rice root tissue was spiked with 1 ng/g of each of the three SLs and used for analysis. Methyl2-{[(3-methyl-5-oxo-2,5-dihydrofuran-2-yl)oxy]methyl}-3-oxocyclopentane-1-carboxylate (GR24) solution served as the internal standard (IS). The application of an internal standard can partially compensate for variations and systematic errors during the analytical process. As showed in Figure 1, GR24 is a synthetic SL analog, is more stable than other SLs, and is commonly used as an internal standard for SL analysis [23]. To correct for potential variations in sample preparation and instrument response, the peak area ratio of the analyte to the internal standard was employed.

#### 2.1.1. Selection of Dispersant and Dispersant Volume

**Figure 2:**
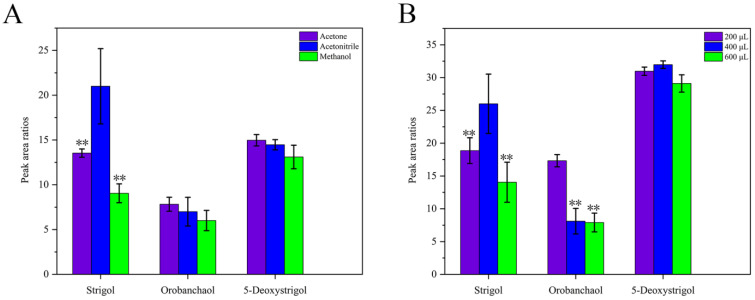
The effect of different dispersant (**A**). The effect of dispersant (acetonitrile) volume (**B**). The peak area ratios refer to the ratios of the peak area of the target analyte to that of GR24 (IS). ** denotes significant differences as determined using the Kruskal–Wallis test with the Dunnett type of contrast (*p* < 0.01).

A ternary system including water, the dispersive solvent, and the extraction solvent was formed in the DLLME-SFO procedure. In typical DLLME-SFO studies, the target analytes are dissolved in an aqueous solution, which necessitates the optimization of both the dispersive and extraction solvent volumes, while maintaining a constant water volume [24]. The efficacy of various solvents (methanol, acetonitrile, and acetone) was evaluated. As shown in Figure 2A, the extraction efficiencies of acetonitrile and acetone for orobanchol, 5-DS, and GR24 were similar, but acetonitrile demonstrated significantly higher efficiency for strigol compared to the other three solvents. This could have been related to factors such as the polarity, volatility, or molecular structures of the solvents as acetonitrile may better dissolve or stabilize strigol during extraction. Therefore, ACN was selected as the preferred dispersant for further experiments.

The dispersant volume is another important factor that affects the extraction efficiency in DLLME-SFO. The impacts of different ACN volumes (200, 400, and 600 µL) on extraction performance were investigated. As shown in Figure 2B, the extraction efficiencies of the 200 µL and 400 µL volumes of acetonitrile were both higher than that of 600 µL. When the extraction volume was 400 µL, the extraction efficiencies for the three compounds were higher than those for 200 µL. Considering the extraction efficiencies of most compounds, 400 µL of ACN was selected for subsequent experiments.

#### 2.1.2. Effect of Ultrasonication Time

**Figure 3:**
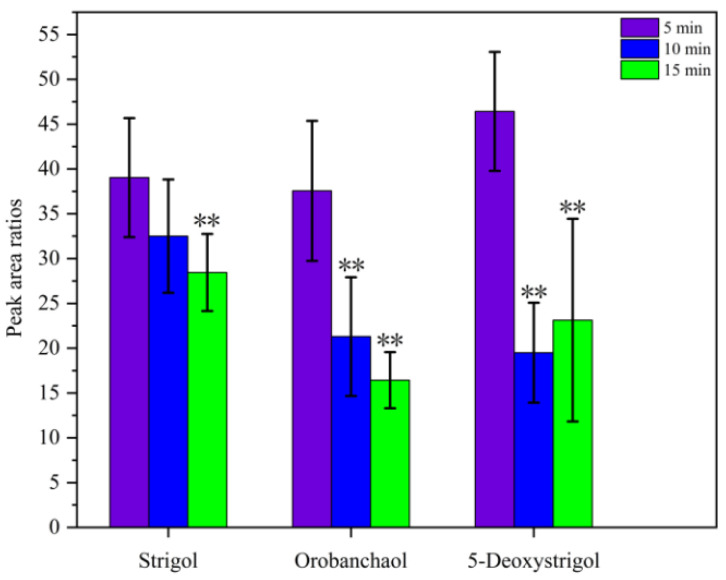
The effects of different ultrasonication times. The peak area ratios refer to the ratios of the peak area of the target analyte to that of GR24 (IS). ** denotes significant differences as determined using the Kruskal–Wallis test with the Dunnett type of contrast (*p* < 0.01).

It has been well established that ultrasonic radiation can facilitate the transfer of analytes to the liquid phase through alternating acoustic pressure [25]. This enhances the efficiency of the extraction process and reduces the overall extraction time [26]. The effect of varying ultrasonication times, from 5 to 15 min, was studied, and the data presented in Figure 3 indicate that a 5 min ultrasonication time was sufficient to complete the extraction. Therefore, a 5 min ultrasonication time was selected for subsequent experiments. Ultrasonic activation aids in enhancing DLLME-SFO extraction efficiency as ultrasound-induced cavitation promotes the formation of well-dispersed solutions and accelerates the mass transfer of analytes from the aqueous phase into the extraction solvent droplets [27].

#### 2.1.3. Selection of Extractant and Extractant Volume

The properties of the extractant have a significant impact on the DLLME-SFO extraction efficiency. The extractant should effectively dissolve the analytes in the aqueous phase, possess favorable chromatographic characteristics, and not interfere with the target analyte. Additionally, the extractant should have a lower melting point than that of the pure analytes. Three hydrophobic extractant (1-undecanol, 1-dodecanol, and n-hexadecane) were evaluated as candidate extractants. The characterization data of the three extractants are presented in Section S1 and Table S1.

**Figure 4:**
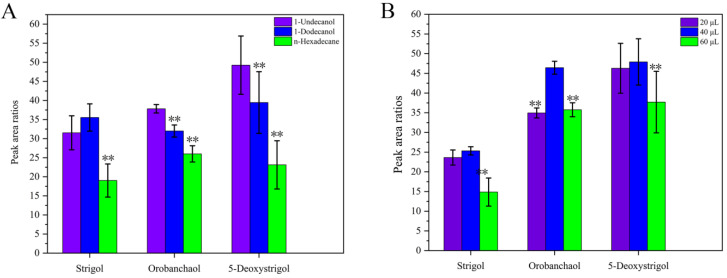
The effects of different extractants (**A**). The effect of extractant (1-undecanol) volume (**B**). The peak area ratios refer to the ratios of the peak area of the target analyte to that of GR24 (IS). ** denotes significant differences as determined using the Kruskal–Wallis test with the Dunnett type of contrast (*p* < 0.01).

As shown in Figure 4A, the extraction efficiency of n-hexadecane was significantly lower than those of 1-undecanol and 1-dodecanol. 1-undecanol exhibited higher extraction efficiency for the three SLs and GR24 compared to 1-dodecanol while 1-dodecanol showed higher extraction efficiency for strigol than 1-undecanol. Based on the principle that compounds with similar polarity tend to be more soluble in each other, 1-undecanol was selected as the extractant for SLs, given its low polarity and ability to efficiently extract these compounds. SLs, typically low-polarity plant hormones, exhibit high solubility in 1-undecanol, making it an effective solvent for extraction. Furthermore, 1-undecanol has the unique property of transitioning into white solid spheres when the environmental temperature falls below its melting point of 16 °C. This phase change aids in separating the extractant from the solution as 1-undecanol, having a lower density than water, forms a solid layer that floats on the surface. The use of 1-undecanol as an extractant offers operational simplicity as it can be easily collected by spooning off the solidified phase at temperatures below its melting point. To ensure a cost-effective and environmentally friendly extraction process, it is crucial to minimize the consumption of 1-undecanol while maintaining the efficient recovery of SLs. The best tested conditions for parameter selection will not only reduce the environmental impact but also improve the overall sustainability of the extraction procedure. Therefore, 1-undecanol was selected for further experiments.

As shown in Figure 4B, the effect of volume on extraction efficiency was investigated by varying the volumes of 1-undecanol. When the volume of the extractant 1-undecanol was 40 µL, the extraction efficiency was higher than that at 20 µL. Additionally, the extraction efficiency at 40 µL was also greater than that at most 60 µL volumes. Based on these results, 40 µL of 1-undecanol was selected for subsequent experiments.

#### 2.1.4. Effect of pH

**Figure 5:**
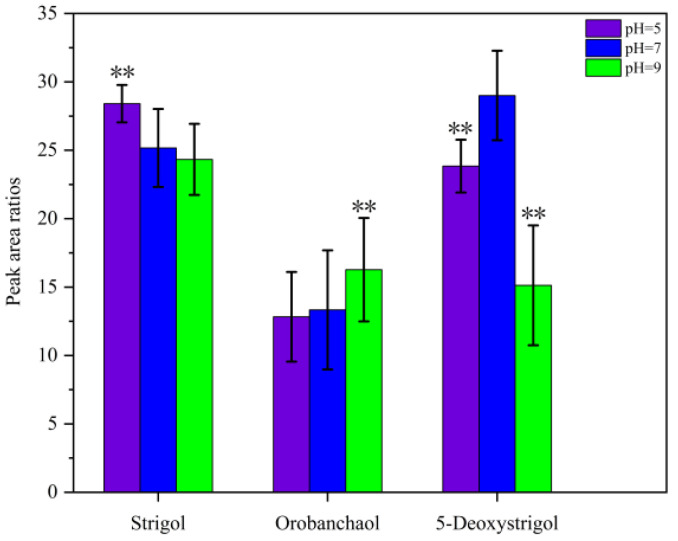
The effect of different pH of the sample solution. The peak area ratios refer to the ratios of the peak area of the target analyte to that of GR24 (IS). ** denotes significant differences as determined using the Kruskal–Wallis test with the Dunnett type of contrast (*p* < 0.01).

The pH of a sample solution can affect the charge state of the analytes and the distribution coefficient between the two phases, thereby influencing their solubility in the extraction solvent [28,29]. The extraction efficiency of the SLs was evaluated by adjusting the pH of the water sample. The results shown in Figure 5 indicated that the extraction efficiency of the SLs remained relatively constant across the pH range of 5 to 9. A pH of 5 was selected for the subsequent experiments.

### 2.2. Method Performance

Under optimal DLLME-SFO and mass spectrometer conditions, the analytical performance of this method was evaluated, including the linearity, coefficient of determination (R^2^), recovery, inter-day precision, intra-day precision, limit of detection (LOD), limit of quantitation (LOQ), and matrix effect. The method performance parameters were as shown in Table 1.

Good linearity was achieved, with coefficient-of-determination (R^2^) values greater than 0.9993. LOD values were in the range of 0.6–1.2 pg/g in rice. LOQ values were in the range of 1.3–6.5 pg/g in rice, indicating the high sensitivity of this method. Recoveries were in the range of 83–96% for SLs. Precision was evaluated using quality control (QC) samples spiked at two concentration levels (1 and 5 ng/g). Both intra-day and inter-day values were assessed to determine the method’s consistency under the same and varying conditions, respectively. The intra-day and inter-day precisions were in the range of 4.5–12.4%, indicating the good precision of the established method. Detailed results of the one-way ANOVA (time) are presented in Supplementary Section S2 and Table S2. No significant differences were observed between inter-day and intra-day precision, indicating that the method was stable and reliable.

The matrix effect was a crucial factor in evaluating the purification capacity of the sample pretreatment method. It reflected the influence of interferences and impurities in the sample matrix on the detection of the target analyte after pretreatment. As shown in Table 1, the matrix effects ranged from 11.3% to 19.3%, reflecting the observed low matrix effects in our study.

### 2.3. Application of the Proposed Method to Rice Samples

**Figure 6:**
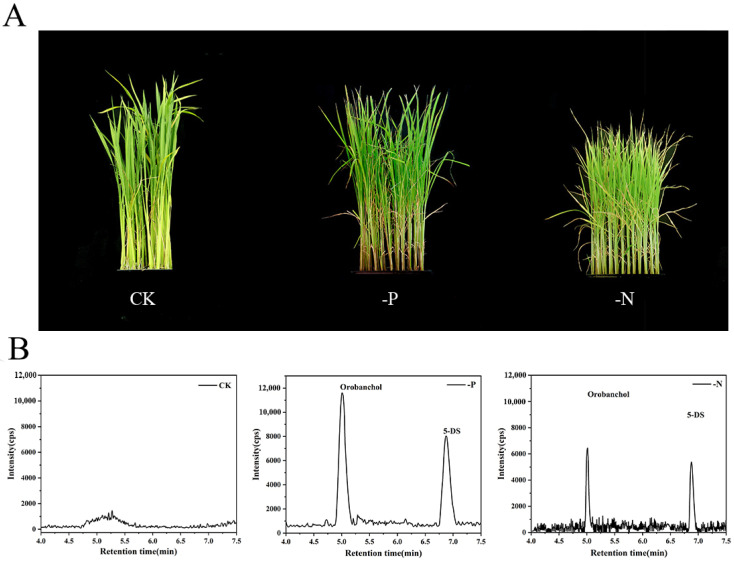
The quantification of 3 SLs in rice under nutrient starvation conditions. (**A**) Rice phenotype. (**B**) MRM chromatogram of targetable SLs in CK samples, -P samples, and -N samples. CK represents samples grown under standard condition; -P represents phosphates starvation samples; -N represents nitrogen starvation samples. The extracted ion chromatograms were obtained using the quantitative ions, which were selectively chosen as specific precursor-product ion pairs.

As previously described by Sun’s work, N/P starvation stimulates the biosynthesis of SLs [30]. The established method was employed to investigate the response of SLs in rice under nitrogen (N) and phosphorus (P) nutrient starvation conditions. SLs, as systemic signals, play a key role in regulating shoot growth in response to phosphorus availability. Additionally, SLs are involved in regulating the levels of phosphate transporters, thereby modulating the efficiency of phosphate uptake under nutrient-deficient conditions [31]. The rice seedlings, 1 week old, were subjected to N/P starvation for 2 weeks. The rice phenotype is shown in Figure 6A. Under N/P starvation, rice seedling leaves exhibit obvious chlorosis. N starvation typically causes chlorosis in the whole plant or lower leaves [32] while P starvation mainly results in chlorosis in the older or lower leaves of rice seedlings [33]. In addition, the leaves of rice seedlings also show morphological changes such as leaf curling and wilting. Figure 6B shows the MRM chromatogram of target SLs in three type (CK,-P, and -N) rice samples. The complete results from the MRM chromatogram of target SLs have been included in Supplementary Section S4 and Figure S1. In Figure S1, the peak of GR24, as the internal standard, was too high, so we have chosen to display only the elution window of the target compounds, which provides a clearer visual representation of the differences in SLs under different treatments.

These results revealed that the concentration of SLs in rice root tissue increases in response to phosphorus and nitrogen deficiency compared to normal growth conditions. This results emphasize the importance of accurately measuring SLs in rice under N/P nutrient starvation conditions as SLs are crucial signaling molecules involved in regulating both the root architecture and nutrient uptake in rice. The significant accumulation of SLs under P deficiency supports the hypothesis that SLs mediate the plant’s response to nutrient stress. It may influence both internal signaling pathways and interactions with soil microorganisms. The ability to quantitatively assess SLs provides a powerful tool for dissecting the molecular mechanisms underlying nutrient sensing and signaling in rice. Understanding how SLs regulate plant growth and nutrient acquisition under N/P stress could inform strategies for improving crop resilience in nutrient-poor soils.

As shown in Table 2, the quantification of SLs in rice under nutrient stress provides valuable insights into the plant’s adaptive mechanisms. In this study, 5-DS was detected at 15.5 ±4.4 pg/g (n = 3) and orobanchol at 10.6 ±6.3 pg/g (n = 3) in the root tissues of rice plants subjected to phosphorus stress for three weeks. Additionally, 5-DS was found at 10.8 ±2.8 pg/g (n = 3) in the root tissues of rice plants under N stress for the same period. These findings corroborate previous reports suggesting that N/P starvation stimulates the biosynthesis of SLs. Notably, orobanchol and 5-DS are the predominant SLs identified in rice, with higher levels of both compounds observed under phosphorus starvation conditions.

The analytical method employed in this study offers a robust tool for the precise measurement of SLs in plant tissues, advancing our understanding of their role in regulating plant responses to nutrient stress. These results underscore the importance of SLs as both plant hormones and signaling molecules, highlighting their potential for further exploration in research on plant nutrient signaling and growth regulation. The ability to accurately quantify SLs opens new avenues for studying their mechanistic roles in plant adaptation to nutrient-limited environments. Additionally, the quantitative analysis method for SLs developed in this study has been validated. The method was also employed to detect 5-DS and orobanchol during the growth of rice under nitrogen or phosphorus nutrient starvation.

Studies have demonstrated that genes associated with the SL signaling pathway, such as *D14*, *D3*, and *D53*, play crucial roles in regulating tiller number in rice [34]. In the breeding of high-yield rice varieties, superior alleles of SL biosynthesis genes, such as *HTD1/D17*, promote increased tiller numbers, thereby enhancing biomass and yield [35].

Additionally, the synthesis of SLs in plants is influenced by environmental factors. For instance, under phosphorus-sufficient conditions, the expression of *NSP2* and its homologs is downregulated, which inhibits SL biosynthesis [17]. Conversely, under low-phosphorus conditions, SL biosynthesis is induced, leading to a suppression of tillering. However, the mechanisms underlying SL action under low-phosphorus stress remain incompletely understood.

This work provides valuable insights for improving crop architecture and supporting the molecular breeding of rice, aiming for reduced fertilizer usage and enhanced yield.

### 2.4. Comparison of DLLME-SFO with Other SL Extraction Methods

In Table 3, a comparison of the DLLME-SFO method with previously reported extraction techniques for SLs is provided. The LOD achieved by the developed method was comparable to or lower than those of other extraction techniques [36,37,38,39]. The method demonstrated high recovery rates ranging from 83% to 96%, indicating the efficient extraction and concentration of SLs from rice root samples [39]. Given the inherent instability of SLs, which renders them susceptible to degradation under light, heat, and hydrolytic conditions, as well as the complex composition of plant matrices, the DLLME-SFO method was optimized for SL extraction/enrichment in rice root samples. This technique utilizes a streamlined, low-temperature solidification step to minimize analyte degradation while achieving superior detection sensitivity (0.6–1.2 pg/g) and recovery rates (83–96%), making it particularly suitable for labile compounds in intricate biological systems.

Traditional solid-phase extraction is time-consuming and costly while other liquid–liquid extraction methods require a large volume of organic solvents, such as ethyl acetate, leading to high solvent consumption. Ethyl acetate is a moderately toxic and highly flammable solvent that may cause irritation to humans and poses environmental concerns due to its volatility and potential to affect the air and aquatic quality. Furthermore, 1-undecanol, the solvent employed in this study, is recognized as non-toxic and environmentally benign compared to alternative methods. Notably, its required volume is as low as 40 µL, enhancing both sustainability and cost-effectiveness. Conventional off-line solid-phase extraction methods typically require long processing times, with vacuum concentration further exacerbating the loss during the process. The methods applied GR24 as an internal standard to compensate for signal loss during sample preparation and matrix interference, thereby reducing reliance on high-purity samples. Compared to existing SL determination methods, the developed method offers improved sensitivity, simplicity, and eco-friendliness.

The classical SL extraction method is liquid–liquid extraction (LLE). LLE employs water-immiscible solvents to extract analytes from aqueous solutions. This is usually accomplished by shaking and collecting the solvent layer containing the analytes of interest. For example, the root exudates were partitioned with ethyl acetate (EtOAc) and 0.2 M K_2_HPO_4_ solution to obtain a neutral EtOAc fraction [40].

**Figure 7:**
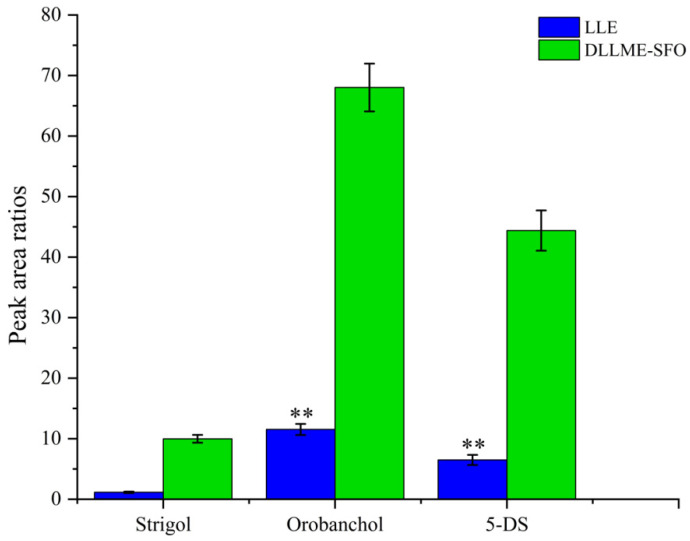
Comparison of sample preparation techniques including DLLME-SFO and LLE on target analyte (SLs spiked at 50 ng/mL). The peak area ratios refer to the ratios of the peak area of the target analyte to that of GR24 (IS). ** denotes significant differences as determined using the Kruskal–Wallis test with the Dunnett type of contrast (*p* < 0.01).

As illustrated in Figure 7, compared with LLE, the DLLME-SFO method yielded significantly greater peak area ratios, indicating improved extraction efficiency. The recovery and matrix effect of the proposed method compared with the conventional LLE method are presented in Section S3 and Table S3 (Supplementary Information), with notable improvements observed particularly for orobanchol and 5-deoxystrigol.

## 3. Materials and Methods

### 3.1. Chemicals and Standards

Three SL standards, strigol, orobanchol, 5-DS, and GR24, were purchased from OlChemIm Ltd. (Olomouc, Czech Republic). GR24 was used as internal standard (IS). Methanol, acetone, acetonitrile, and ethyl acetate (EtOAc) (HPLC-grade) were purchased from Sigma-Aldrich (Sigma-Aldrich, St. Louis, MO, USA). 1-undecanol (purity > 98%), 1-dodecanol (purity > 98%), and n-hexadecane (purity > 98%) were purchased from Aladdin (Shanghai, China). Ultrapure water (resistivity ≥ 18.25 MΩ/cm) obtained from WaterPro water system (ULUPURE, Chengdu City, Sichuan Province, China) was used in all experiments. Individual stock standard solutions (1000 mg/L) of each compound were prepared using methanol and stored in the freezer at −20 °C. The mixed standard solutions were stored at 4 °C. All the reagents were of analytical grades (least 98% purity).

### 3.2. Instrument Analysis

Chromatographic analysis of the targeted SLs was conducted using a Thermo Scientific™ Vanquish™ UHPLC. Separation was achieved on an Acquity UHPLC BEH C18 column (1.7 µm, 2.1 × 50 mm, Waters, MA, USA) maintained at 40 °C. Mobile phase A consisted of 0.1% (*v*/*v*) formic acid in water, and mobile phase B was methanol, with a flow rate of 0.3 mL/min. The injection volume was 5 µL. The elution program was as follows: 0 min: 25% B; 1 min: 30% B; 2 min: 40% B; 3 min: 50% B; 4 min: 60% B; 5 min: 70% B; 6 min: 80% B; 7 min: 90% B; 8 min: 100% B; 9 min: 25% B; 14 min: 25% B.

Mass detection was performed using a TSQ Quantis triple quadrupole mass spectrometer (Thermo Fisher Scientific, Waltham, Massachusetts, USA) equipped with an electrospray ionization (ESI) source operating in negative mode. Mass spectrometry parameters—ion source voltage: ESI at 2800 V; negative ion scanning; detection mode: Multiple Reaction Monitoring (MRM); ion transfer tube temperature: 325 °C; sheath gas (N_2_) pressure: 40 Arb; auxiliary gas (N_2_) pressure: 10 Arb.

**Figure 8:**
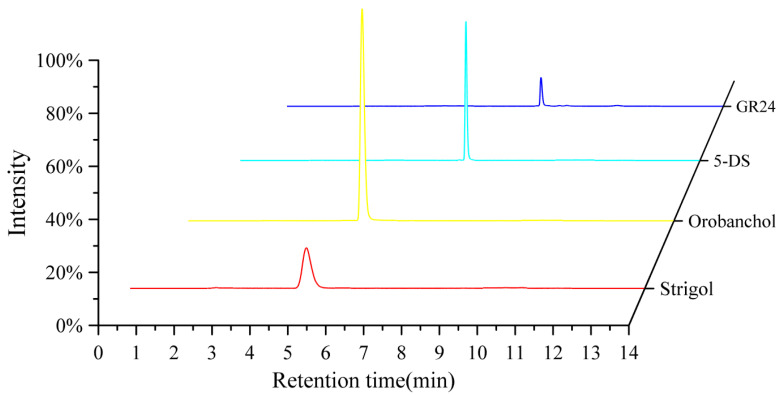
Liquid chromatography diagram of three SLs and GR24 in the standard sample at 50 ng/mL.

As shown in Figure 8, the results demonstrated that the analytes exhibited excellent separation and distinct fragmentation patterns. The MRM parameters of the SLs are summarized in Table 4.

### 3.3. Plant Material and Growth Conditions

Rice (Nipponbare) was cultivated in a greenhouse at the Institute of Hunan Provincial Key Laboratory of Phytohormones and Growth Development (Changsha City, China).

Plump seeds were selected and placed in Petri dishes. The seeds were soaked in distilled water for germination induction and maintained at 30 °C in a constant-temperature incubator for 48 h. Upon radicle emergence (germination), the sprouted seeds were transferred to rice cultivation containers filled with nutrient solution for subsequent growth. The rice was cultured at 28 °C in a greenhouse under a 14 h light/10 h dark photoperiod.

After one week of normal culture with Kimura B nutrient solution, rice plants were transferred to a P-deficient Kimura B nutrient solution for the phosphorus (P) starvation treatment. For nitrogen (N) starvation treatment, the plants were cultured with a N-deficient Kimura B nutrient solution for three weeks. Rice root tissues were then collected after the respective treatments. The tissue samples were fine-ground in a liquid nitrogen environment. Following the optimized sample pretreatment steps outlined in this study, the contents of the three SLs in the rice root tissues from the CK, -P, and -N treatment groups were analyzed. All rice root samples were immediately frozen in liquid nitrogen and stored at −80 °C for later use.

### 3.4. DLLME-SFO Procedure

**Figure 9:**
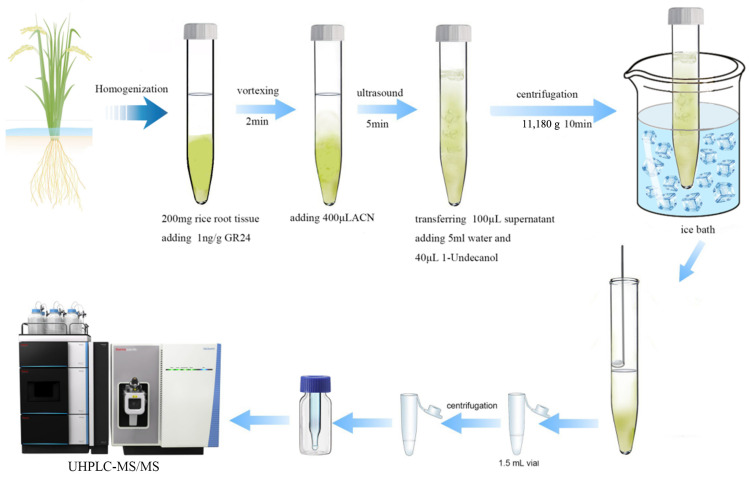
The sample pretreatment procedure.

A quantity of 200.0 mg of the rice root tissue was ground and transferred into a 10 mL centrifuge tube. GR24 (IS) was then added to the tube and the tube was held for 30 min to allow GR24 to integrate well into the rice root sample. Then, 400 µL of acetonitrile, an organic solvent, was added for extraction, followed by sonication and centrifugation (11,180× *g* for 10 min). The 100 µL supernatant was employed as the dispersing agent, and 1-undecanol was added as the extracting agent, along with water. The mixture was vortexed, homogenized, and then ice-bathed. After the sample had solidified and been frozen, it was extracted with a spatula (or scoop) and transferred into a sample vial containing an insert. The sample was subsequently analyzed after the 1-undecanol dissolved at room temperature. The general process of sample pretreatment is illustrated in Figure 9.

### 3.5. Method Validation

The rice matrix blank samples and solvent blank samples were prepared in every set of samples. With three replicates per experiment. After every 15 samples, the quality control (QC) samples were evaluated including matrix blank samples, solvent blank samples, and spiked rice samples (1 ng/mL). The continuing calibration verification standard solution and continuing calibration blank were used to verify the UHPLC-MS/MS drift.

To validate the developed method, the linearity, LODs, LOQs, precision, and matrix effect (ME) were investigated. Calibration samples at 7 concentrations (0.1, 0.5, 1, 5, 10, 20, and 50 ng/mL of SLs) with a fixed concentration of IS (GR24 1 ng/g) were used. To evaluate sensitivity, the method LODs were defined as the concentrations corresponding to a signal-to-noise ratio of 3, and the method LOQs were defined as the concentrations corresponding to a signal-to-noise ratio of 10, based on the lowest spiked concentration (0.10 ng/mL). The trueness of the method was evaluated based on the recovery of QC samples spiked with analytes at both low and high concentrations. Intra-day precision and inter-day precision were assessed to determine the method’s performance under consistent and variable conditions, respectively. The intra-day precision was evaluated by analyzing 7 replicates on the same day while the inter-day precision was assessed over five consecutive days (7 replicates per day). Given the inherent complexity of rice root material, further investigation was conducted to assess the potential for interference with the analytical process.

The recoveries of 3 SLs in rice were evaluated by spiked standards of low (1 ng/g) and high (5 ng/g) concentrations. Recovery (%) quantifies the efficiency of an analytical method to accurately measure a known quantity of analyte added to a sample. The recovery value was then calculated using Equation (1).

Recovery (%) = (C _measured_ − C _unspiked_/C _added_) × 100% (1)

The response value of SLs obtained by spiking with a pure solvent and prior pretreatment was compared to the response value of SLs obtained by spiking in the sample matrix with prior pretreatment. The matrix effect (ME) value was then calculated using Equation (2). It is calculated by comparing the response of an analyte in a post-extraction spiked matrix to its response in a pure solvent (absence of matrix) [41].

ME (%) = (Slope _spiked matrix_/Slope _pure solvent_) × 100% (2)

## 4. Conclusions

This study employed a rapid and eco-friendly DLLME-SFO method combined with UHPLC-MS/MS technology for the separation and sensitive detection of SLs.

SLs exhibit high physiological activity, but they are unstable and sensitive to temperature. The one-step low-temperature solidification process, which includes extraction and enrichment of SLs from rice root samples using DLLME-SFO, enhances sensitivity and simplifies the process. This method has been successfully applied for the simultaneous determination of three SLs in rice. DLLME-SFO is rapid because it eliminates multi-step conditioning/phase separation, reducing the preparation time compared to LLE/SPE. It is eco-friendly due to its lower solvent consumption, use of low-toxicity solvents, and alignment with green chemistry metrics. These advantages have been quantitatively validated in complex matrices like rice, making the method both technically superior and environmentally sustainable. The validated analytical method provides a valuable tool for a wide range of research on SLs. It facilitates not only a deeper understanding of their mechanistic roles as plant hormones but also offers new opportunities to explore their functions as signaling molecules in plants. This methodological advancement is essential for advancing our knowledge of SLs’ involvement in plant growth regulation and stress responses.

## Figures and Tables

**Table 1:** Results of method performance and validation study.

Analytes	Equation of Linear Regression	Linear Range (ng/mL)	R^2^	Recovery (%)		Inter-Day Precision (RSD%)		Intra-Day Precision (RSD%)		LODs (pg/g)	LOQs (pg/g)	Matrix Effects(%)
				Low (1 ng/g)	High (5 ng/g)	Low (1 ng/g)	High (5 ng/g)	Low (1 ng/g)	High (5 ng/g)			
Strigol	y = 3.30x + 0.9144	0.1–50	0.9993	83	89	8.9	11.4	8.3	6.7	1.2	6.5	111.3
Orobanchol	y = 2.11x + 0.1019	0.1–50	0.9995	90	91	10.2	6.3	11.4	8.6	0.8	3.5	115.2
5-Deoxystrigol	y = 1.14x + 1.3491	0.1–50	0.9996	96	93	11.7	5.6	12.4	4.5	0.6	1.3	119.3

**Table 2:** SL content in rice root tissue after 3 weeks of normal culture and 1 week of -N/-P treatment. Each treatment consisted of 50 plants.

Analytes	CK (pg/g)	-P (pg/g)	-N (pg/g)
Strigol	N.D.	N.D.	N.D.
Orobanchol	N.D.	10.6 ± 6.3	8.8 ± 3.5
5-Deoxystrigol	N.D.	15.5 ± 4.4	10.8 ± 2.8

Note: N.D.—not detected (below minimum detection limit of the method). The uncertainty was estimated directly as the standard deviation of replicate measurements.

**Table 3:** Comparison of the developed method with other methods used for preconcentration and determination of SLs.

Sample Source	SLs	Extraction Method	Extraction Solvent	LOD	Recovery (%)	Matrix Effect (%)	Reference
Pea (*Pisum sativum*)	orobanchol, orobanchyl acetate, and fabacyl acetate	LLE	ethyl acetate	0.14–4.94 μg/L	57.3–77.0	66.4–162.1	[36]
Tomato (*Solanum lycopersicum*)	7-oxoorobanchyl acetate, solanacol, orobanchol, strigol, fabacyl acetate, orobanchyl acetate, and 5-DS	LLE	ethyl acetate	0.02–0.96 μg/L	80.2–108.1	Not explicitly mentioned	[37]
Sorghum (*Sorghum bicolor*), rice (*Oryza sativa*), pea (*Pisum sativum*), and tomato (*Solanum lycopersicum*)	strigol, solanacol, orobanchol, sorgomol, GR24, sorgolactone, 4-DO, 5-DS, carlactonoic acid, carlactone	Rapid extraction with organic solvents (e.g., acetone) followed by single-step preconcentration on polymeric RP SPE sorbent	5% acetonitrile/water (*v*/*v*) for root exudates, 60% acetone/water (*v*/*v*) for root tissues	0.125–2.5 fmol	83.1–95.6	61.8–102.5	[38]
Rice (*Oryza sativa*)	5-DS	Tailored solid-phase extraction (SPE) procedure based on physicochemical properties	90% methanol	0.29 pg	45.6–70.5	89.1–96.7	[39]
Rice (*Oryza sativa*)	Strigol, 5-DS, Orobanchol	DLLME-SFO	1-undecanol	0.6–1.2 pg/g	82.6–96.3	111.3–119.3	This work

**Table 4:** The MRM parameters of the SLs.

Analytes	RT (min)	Parent Ion(*m*/*z*)	Product Ion (*m*/*z*)	Collision Energy(eV)	RF Lens (V)
Strigol	4.88	369.23	272.05 */257.14	15.87/24.29	169
Orobanchol	5.01	347.25	314.96 */328.94	9.00/10.01	142
5-Deoxystrigol	6.87	331.24	216.18 */97.03	16.20/20.71	118
GR24(IS)	8.15	321.18	223.91 */303.00	14.48/11.95	184

Note: * quantitative ions; RF lens: radiofrequency lens.

## Data Availability

The data presented in this study are available on request from the corresponding author.

## Supplementary Materials

The supporting information can be downloaded at: https://www.mdpi.com/article/10.3390/ijms26094337/s1.
