# Supplementary material for: Development of Dispersive Liquid–Liquid Microextraction Method Based on Solidification of Floating Organic Droplets for Rapid Determination of Three Strigolactones in Rice (Oryza sativa L.) Using Ultra-High-Performance Liquid Chromatography–Tandem Mass Spectrometry
Source: Int J Mol Sci. 2025 May 2;26(9):4337. doi: 10.3390/ijms26094337 (PMC12072799; doi:10.3390/ijms26094337)
Supplement: Supplementary file 1 [file ijms-26-04337-s001.zip › ijms-3526737-supplementary.pdf]

Supplementary Information

Article

Development of dispersive liquid–liquid microextraction method based on solidification of floating organic droplets for the rapid determination of three strigolactones in rice using ultra performance liquid chromatography–tandem mass spectrometry

Xianxin Zhu [1,2], Zihan Wu [1,2], Xunzhi Deng[1,2], Ze Liao[1], Ruozhong Wang *[1,2], Zhoufei Luo *[1,2]

1 College of Bioscience and Biotechnology, Hunan Agricultural University, Changsha, 410128, China

2 Hunan Provincial Key Laboratory of Phytohormones and Growth Development, Hunan Agricultural University, Changsha, China

* Correspondence: wangruozhong@hunau.edu.cn (R.Z . Wang); zhoufeiluo@hunau.edu.cn(Z.F. Luo)

Section S1: The Best Tested Conditions of the DLLME-SFO parameters

The characteristics of the extractant play a crucial role in determining the efficiency of DLLME-SFO. An ideal extractant should efficiently solubilize the analytes from the aqueous phase, exhibit suitable chromatographic behavior, and remain free of interference with the target compounds. Furthermore, its melting point should be lower than that of the pure analytes.Three hydrophobic extractant (1-undecanol, 1-dodecanol, and n-hexadecane) were evaluated as candidate extractants. The characterization of three extractants was summarized in Table S1.

Section S2: ANOVA to Evaluate the Effects of Time (Inter-day and Intra-day)

As shown in Table S2, ANOVA was used to evaluate the effects of time. No statistically significant differences were observed among inter-day and intra-day measurements for all three compounds at both low and high concentrations ($p > 0.05$), demonstrating the high stability and reliability of the analytical methods.

Section S3: Comparison of sample preparation techniques including DLLME-SFO and LLE on target analyte on recovery and matrix effect (n=3)

Comparison of sample preparation techniques including DLLME-SFO and LLE in Table S3.A comparison of recovery and matrix effects between the proposed method and the conventional LLE approach revealed that the proposed method exhibited improved performance in both parameters.

Section S4: The MRM chromatogram of targetable SLs in CK samples, -P samples, -N samples (n=3)

Given the relatively low abundance of SLs in rice roots and the high signal intensity of the internal standard GR24 in Figure S1, the chromatographic peaks in the CK group were not clearly visible. To better illustrate the treatment-specific differences, chromatograms for CK, –P, and –N conditions were individually displayed in Figure 6.

**Figure and table captions:**	
**Table S1.**	Characterization of three candidate extractants.
**Table S2.**	ANOVA to Evaluate the Effects of Time (inter-day and intra-day)
**Table S3.**	Comparison of sample preparation techniques including DLLME-SFO and LLE on target analyte (n=3)
**Figure S1.**	The MRM chromatogram of targetable SLs in CK samples, -P samples, -N samples (n=3)

Table S1. Characterization of three candidate extractants.

Candidate extractants	Chemical formula	Molecular weight	Density at 25 °C (lit.) g/cm³	Freezing point (°C)	Solubility
1-Undecanol	$C_{11}H_{24}O$	172.3077	0.8300	15.90	insoluble in water, soluble in alcohol
1-Dodecanol	$C_{12}H_{26}O$	186.3342	0.8310	23.95	insoluble in water, soluble in ethanol
n-Hexadecane	$C_{16}H_{34}$	226.4400	0.7734	18.20	insoluble in water, miscible with diethyl ether, petroleum ether and chloroform, slightly soluble in hot ethanol

Table S2. ANOVA analysis effects of different time.

Compound	Concentration (ng/g)	*t*-value	*p*-value	Significance conclusion
Strigol	1	0.07	0.946	No significant difference ($p > 0.05$)
	5	-0.24	0.817	No significant difference ($p > 0.05$)
Orobanchol	1	0.09	0.989	No significant difference ($p > 0.05$)
	5	-0.76	0.689	No significant difference ($p > 0.05$)
5-Deoxystrigol	1	-0.03	0.978	No significant difference ($p > 0.05$)

Compound	Concentration (ng/g)	*t*-value	*p*-value	Significance conclusion
	5	-0.56	0.589	No significant difference (p > 0.05)

Table S3. Comparison of sample preparation techniques including DLLME-SFO and LLE on target analyte (n=3) .

Compound	Sample preparation techniques	Recovery(%) n=3	matrix effect(%) n=3
Strigol	DLLME-SFO	86	116.6
	LLE	63	132.6
Orobanchol	DLLME-SFO	90	112.2
	LLE	68	134.3
5-Deoxystrigol	DLLME-SFO	95	113.3
	LLE	65	142.4

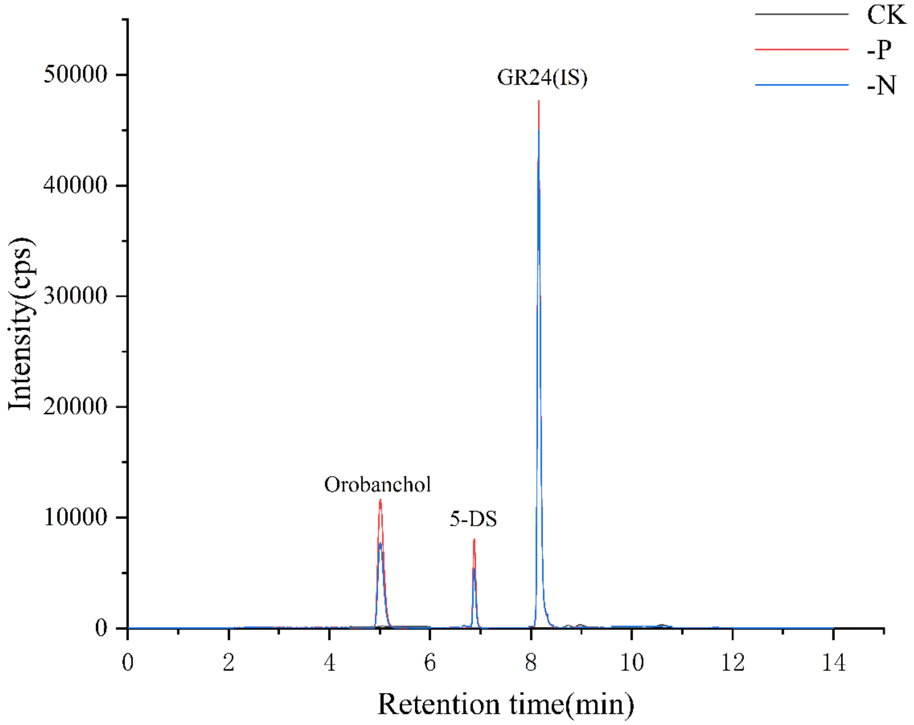

Figure S1. The MRM chromatogram of targetable SLs in CK samples, -P samples, -N samples.CK represents samples grown under standard condition; -P represents phosphates starvation samples; -N represents nitrogen starvation samples. The extracted ion chromatograms were obtained using the quantitative ions (*marked) in Table 4, which were selectively chosen as specific precursor-product ion pairs.
